# Mini-ALPPS-based multidisciplinary treatment leading to long-term survival in a patient with a large HCC: A case report

**DOI:** 10.3389/fsurg.2022.920953

**Published:** 2023-01-24

**Authors:** Gao-Min Liu, Yao-Min Zhang

**Affiliations:** ^1^ Department of Hepatobiliary Surgery, Meizhou People’s Hospital, Meizhou, China; ^2^ Guangdong Provincial Key Laboratory of Precision Medicine and Clinical Translational Research of Hakka Population, Meizhou, China

**Keywords:** HCC, mini-ALPPS, TACE, salvage, long-term survival

## Abstract

**Background:**

The future liver remnant (FLR) induced by stage I associated liver partition and portal vein ligation for staged hepatectomy (ALPPS) in hepatocellular carcinoma (HCC) might be limited due to liver fibrosis/cirrhosis or incomplete liver parenchymal transection.

**Case presentation:**

A 51-year-old male with hepatitis B liver fibrosis was diagnosed with a large HCC (13.5 cm × 12.5 cm × 13.8 cm). The FLR of the patient was insufficient to permit one-stage tumor resection. Therefore, the two-stage ALPPS surgery was planned. Stage I ALPPS was performed with incomplete liver parenchymal transection due to bleeding (which is why we called it Mini-ALPPS). On postoperative day (POD) 18, CT revealed that the FLR hypertrophy was poor. The FLR/standard liver volume (SLV) had only increased from 22.00% to 34.63%. Salvage transhepatic arterial chemoembolization (TACE) was performed on POD 22 days to control possible tumor progression during the waiting period and to further facilitate FLR growth. About 16 days later, a CT reassessment of FLR revealed a 42.5% FLR/SLV. A right hepatectomy was then uneventfully performed. Although HCC recurred after 586 days, the patient survived for more than 1,920 days after stage II ALPPS.

**Discussion:**

Damage control during a difficult conventional stage I ALPPS was important. TACE during the interstage and postoperative periods of this Mini-ALPPS was safe and beneficial. A multidisciplinary based on Mini-ALPPS treatment could provide patients long-term survival; however, Mini-ALPPS should not be selected as the primary solution for such patients today, as some other minimally invasive and effective strategies are available.

## Background

Hepatocellular carcinoma (HCC) remains a major public health challenge worldwide (1). Other than liver transplantation, surgical resection of HCC provides the best oncological outcome. However, most patients are not candidates for radical resection due to inadequate future liver remnant (FLR) or metastasis/progression of HCC at diagnosis (2). Associated liver partition and portal vein ligation for staged hepatectomy (ALPPS), which might induce rapid FLR hypertrophy, can make it impossible to resect HCC in certain patients (3). Notably, most HCC patients suffer from liver fibrosis or cirrhosis, and these conditions may lead to incomplete liver parenchymal transection or poor growth of the FLR after stage I ALPPS. As a result, stage II ALPPS cannot be performed as planned (4).

Here, we present a long-lived case in which a patient with a large HCC was originally planned to receive ALPPS; however, he received Mini-ALPPS as a result of incomplete liver parenchymal transection during stage I surgery. FLR hypertrophy was poor following stage I Mini-ALPPS. Salvage transhepatic arterial chemoembolization (TACE) was then performed. Finally, the FLR met the criteria for a major hepatectomy, and the patient received a right hepatectomy. After 1,922 days of follow-up, the patient is still alive.

## Case presentation

**Figure 1:**
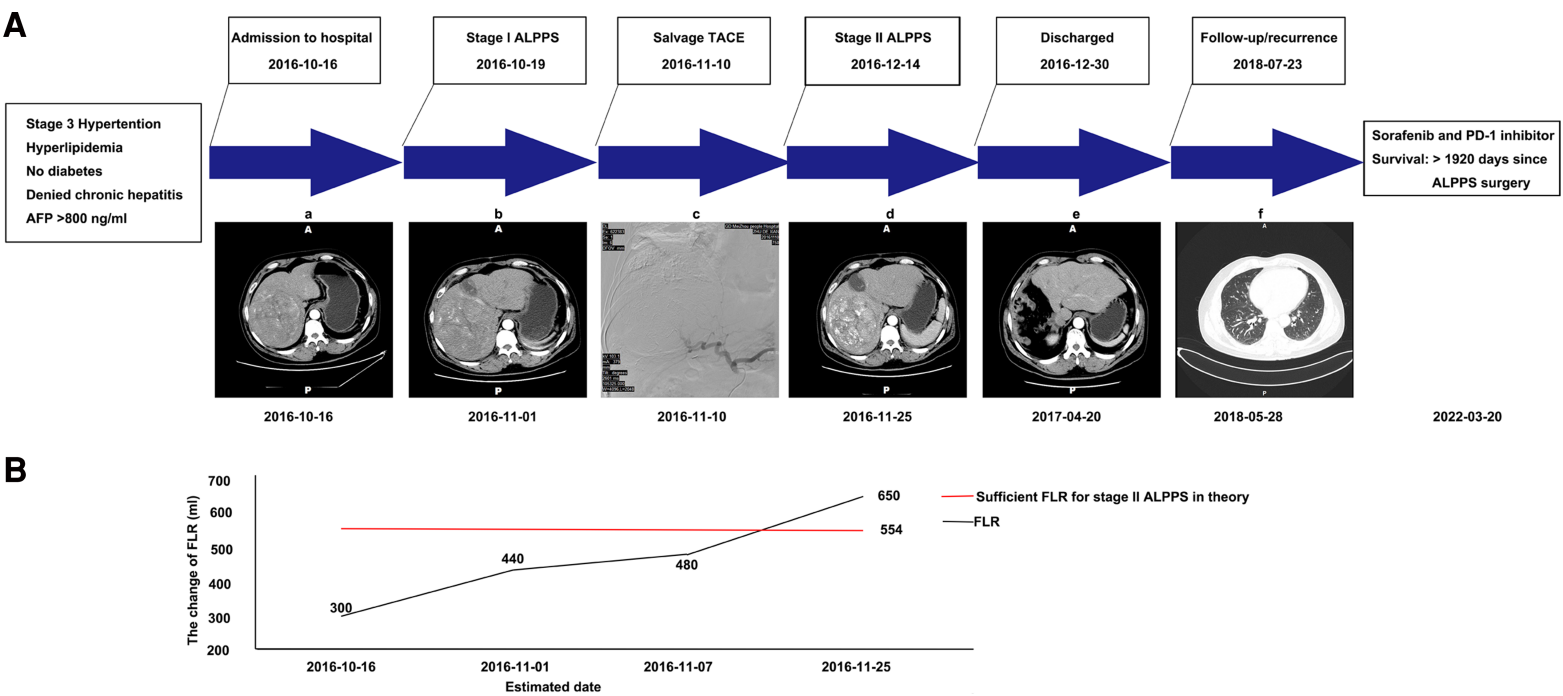
Graphic summary of the case. (**A**) CT scans obtained before stage I ALPPS (a), after stage I ALPPS (b), during TACE (c), after TACE (d), after stage II ALPPS (e), and showing lung metastasis during follow-up (f). (**B**) The change of estimated future liver remnant (FLR). CT, computed tomography; ALPPS, associated liver partition and portal vein ligation for staged hepatectomy; TACE, transhepatic arterial chemoembolization; FLR, future liver remnant.

A 51-year-old male was found to have a large HCC (13.5 cm × 12.5 cm × 13.8 cm) in liver segments 5–8 during a routine check-up. The patient had no clinical symptoms at the time of the diagnosis. The patient was infected with hepatitis B years ago and had stage 3 hypertension and hyperlipidemia, but not diabetes. The body weight, height, and BMI of the patient were 83 kg, 1.71 m, and 28.38 kg/m^2^, respectively. Laboratory tests indicated that the patient's levels of alanine transaminase (ALT; 325 U/L), alkaline phosphatase (ALP; 186 U/L), total bilirubin (TBIL; 40.0 μmol/L), direct bilirubin (DBIL; 10.3 μmol/L), creatinine (155 μmol/L), and blood ammonia (66 μmol/L) were abnormal. The alpha-fetoprotein (AFP) level was significantly increased (>800 ng/ml). The patient's liver function was grade A, with a Child–Pugh score of 6. The preoperative retention rate of ICG at 15 min was not available due to a lack of equipment in our hospital at that time. The HCC had not invaded the left lobe of the liver or adjacent/distant organs in preoperative assessment. The tumor stage was Barcelona Clinic Liver Cancer (BCLC) stage B and China liver cancer stage Ib. The estimated preoperative standard liver volume (SLV) was 1386.16 ml, and the evaluated FLR was 300 ml based on CT assessment (Figure 1). The 22% FLR/SLV was insufficient to permit the removal of the tumor in one operation with major hepatectomy; the risk of posthepatectomy liver failure (PHLF) would be too high (5). After a multidisciplinary team (MDT) discussion, ALPPS-based multidisciplinary treatment was planned, and the patient agreed to receive such treatment.

## Stage I Mini-ALPPS

The surgery was performed laparoscopically. First, the gallbladder was resected. The right portal vein was separated from the hilar plate and clipped with a Hem-o-Lok (Johnson & Johnson, United States). Then, the parenchymal transection was performed *via* an anterior approach, following the ischemia line on the liver. Notably, intraoperative bleeding was difficult to control due to fibrosis and edema. To control the damage, the liver parenchymal tissue was only partially transected (to a point approximately 5 cm from the liver surface). Thus, it would be more appropriate to call our procedure “Mini-ALPPS.” The right hepatic pedicle was sutured with black suture silk (Johnson & Johnson—Ethicon, United States). Finally, a surgical drain (Guangdong Sunlight Medical Ltd., China) was routinely placed in the hepatorenal fossa and the foramen of Winslow. The surgery required approximately 255 min and resulted in the loss of 800 ml of blood. On postoperative day (POD) 18 after stage I Mini-ALPPS, a CT was performed to re-estimate the degree of FLR hypertrophy. After evaluation, the FLR/SLV was only 34.63%; this was still not sufficient for stage II right hepatectomy (5). Based on this finding, we and the patient considered further interventions to facilitate FLR hypertrophy and prevent potential tumor progression during the waiting period.

## Salvage TACE

**Figure 2:**
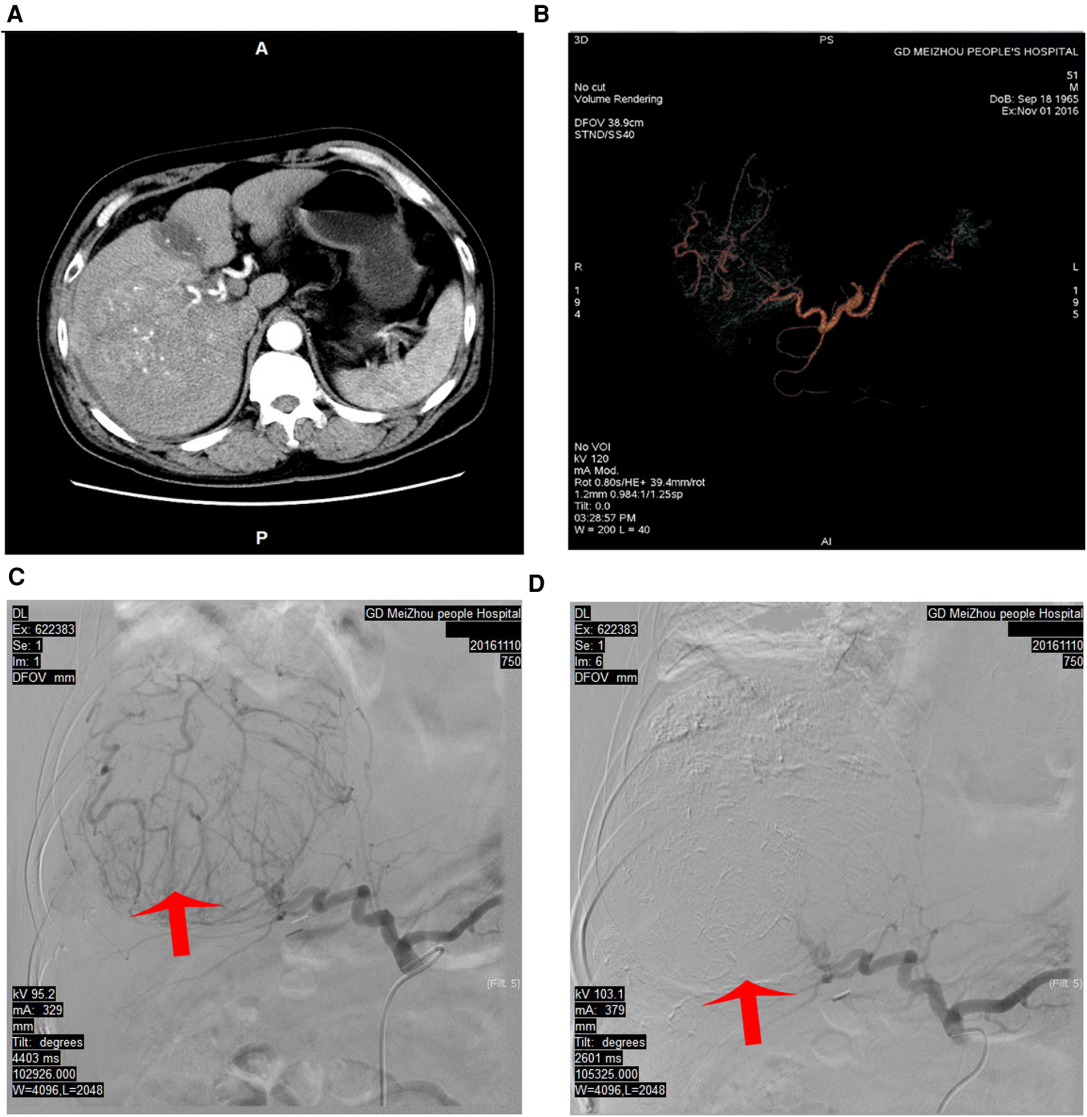
Hepatic angiography and TACE were performed to block the main arterial supply to the tumor. (**A**) The arterial phase of the CT scan before TACE. (**B**) The CT vascular reconstruction before TACE. (**C**) The main arterial blood supply to the tumor and significant tumor staining before TACE. (**D**) The main artery supplying the tumor was blocked using super-selective TACE, and the tumor staining disappeared. TACE, transhepatic arterial chemoembolization; CT, computed tomography.

After stage I surgery, a CT scan still revealed a rich arterial blood supply to the tumor. After an MDT discussion, we decided to administer TACE to the patient. On POD 22 after stage I Mini-ALPPS, TACE was performed. Hepatic angiography showed that the main arterial supply of the tumor originated from the right hepatic artery. Arterial derangement of the large tumor could also be observed. The right hepatic artery was blocked with 10 ml lipiodol mixed with 20 mg epirubicin and 20 mg lobaplatin. After chemoembolization, the arterial flow to the large tumor almost completely disappeared (Figure 2). After stage I Mini-ALPPS, CT on POD 37 revealed that the FLR/SLV had increased to 46.89%. Theoretically, the FLR/SLV now meets the requirements of stage II hepatectomy; however, due to the patient’s moderate fever after TACE, stage II surgery was not performed until POD 56 after stage I surgery.

## Stage II Mini-ALPPS

**Figure 3:**
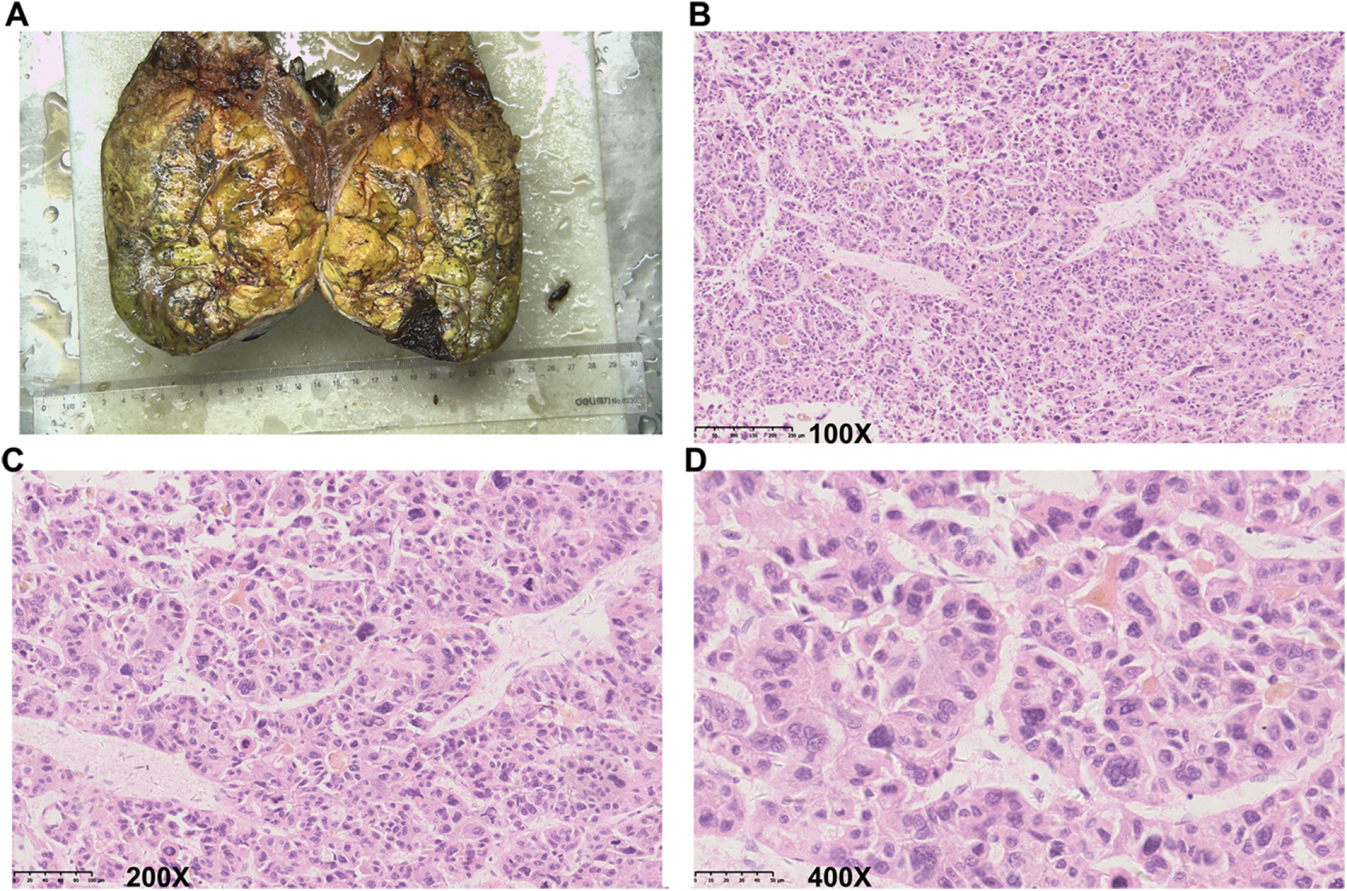
Representative images of the resected tumor and histopathological examination. (**A**) Representative image of the resected tumor. (**B–D**) Representative images show the results of histopathological examination. (**B**) 100× magnification. (**C**) 200× magnification. (**D**) 400× magnification.

Considering the intraoperative bleeding that occurred at stage I, an open operation was planned at stage II to better control possible bleeding. Before the stage II surgery, the AFP level decreased to 126.81 ng/ml. During the surgery, we found significant hypertrophy of the left lobe of the liver. The parenchymal transection was still performed with anterior approaches. The right hepatic artery, right hepatic duct, and right portal vein were successively separated and cut after ligation with Hem-o-Lok (Johnson & Johnson, United States). The remaining liver parenchyma was then gradually dissected using an ultrasonic scalpel (Johnson & Johnson, United States) and bipolar coagulation (KANGJI Medical, China). The major vein of the right liver was separated, cut, and continuously sutured with 5-0 Prolene suture (Johnson & Johnson, United States). The right hemihepatectomy was completed after the removal of all related ligaments of the right liver. Finally, as is routine, a silicone drainage tube (Guangdong Sunlight Medical Ltd., China) was placed into the right subphrenic space. The surgery required approximately 260 min and resulted in the blood loss of 450 ml. One unit of packed red blood cells was administered, and the patient recovered without eventful complications. Histopathology confirmed a 12 cm × 9 cm × 14 cm HCC in the resected liver (Figure 3). The paracancerous liver tissues were graded as G1S1 according to the biopsy criteria of the Chinese Program of Prevention and Cure for Viral Hepatitis (6) and graded as F1 fibrosis according to the METAVIR classification (7). The patient was discharged 15 days after stage II Mini-ALPPS. The patient received adjuvant transarterial chemotherapy 30 days after stage II Mini-ALPPS. 3 months later, his TBIL (40.0 μmol/L), DBIL (10.7 μmol/L), and creatinine (138 μmol/L) were still moderately elevated, but his AFP (3.94 ng/ml) was normal, and the CT scan showed no sign of residual tumor or recurrence. The patient was followed up regularly, every 3 months. After 586 days, stage II Mini-ALPPS, the patient was diagnosed with tumor recurrence (his AFP was 20.18 ng/ml, and his CT scan revealed the presence of two novel subcapsular nodules in the residual left liver and multiple metastatic nodules in the lung). The patient then received sorafenib and PD-1 inhibitor therapy. The patient has survived for 1,920 days since ALPPS surgery so far.

## Discussion

Portal vein embolization (PVE) is the mainstream strategy to modulate FLR for decades. However, the long waiting period (usually >3–4 weeks) of PVE can result in tumor progression before staged hepatectomy (8). Sequential or simultaneous TACE and PVE could help control the tumor progression and further facilitate FLR regeneration, but they did not substantially reduce the long waiting period for staged hepatectomy (9). A combination of PVE and hepatic vein embolization (HVE) significantly shortened the waiting period to 3–4 weeks and tended to be safe and effective (10). ALPPS induces the most rapid hypertrophy of FLR and confers a higher rate of earlier resection compared with PVE-based treatments above, but it is associated with a high postoperative mortality and complications rate. Especially in HCC patients with hepatic fibrosis or cirrhosis, the risk of ALPPS was much higher and the rate of FLR growth was much lower than in non-HCC (11). For patients with mild fibrosis and solitary HCC like this case, PVE-based treatments but not ALPPS could be the primary solution in other centers. However, as PVE-based treatments were not regularly performed in our center at that time, the most common treatment for such patients would be TACE. The long-term prognosis of TACE treatment for such HCC is far from satisfying (12). Based on the experienced hepatectomy techniques in our department and the baseline characteristics of the patient (solitary HCC with mild fibrosis), we finally decided to try ALPPS. But considering the high risk and unclear long-term prognosis of ALPPS, PVE combined with TACE or HVE might be an alternative, safe, and minimally invasive strategy to conventional ALPPS today.

Too much intraoperative bleeding impeded the complete transection of the liver parenchymal at stage I. We did not convert to open surgery to try to achieve standard ALPPS as, on the one hand, the intraoperative bleeding was due to fibrosis and liver edema and hard to control and, on the other hand, massive hemorrhage could also occur in open surgery, which would increase the risk of postoperative complications. To control the damage from stage I surgery, we stopped further transection. Considering the actual percentage of transected liver parenchymal, it would be more appropriate to call our technique “Mini-ALPPS.” Postoperative uneventful recovery of the patient further proved the correctness of our intraoperative decision. The patient's FLR increased from 22% to 46.89% in 37 days. Both the liver fibrosis and mini transection of the liver parenchymal might lead to a relatively long interstage waiting period. From this point of view, this Mini-ALPPS procedure has lost the advantage of rapid hypertrophy of FLR over other FLR modulation strategies.

Salvage interventions are critical in managing inadequate hypertrophy after stage I ALPPS. A rare case was reported in which transhepatic arterial embolization (TAE) was utilized to salvage the failure of stage I ALPPS in a patient with a large HCC, and stage II ALPPS was finally completed in that patient (13). Radiofrequency ablation or percutaneous ethanol injection was also reported to rescue failed stage I ALPPs in patients with cirrhosis-related hepatocellular carcinoma (14). More recently, Zhuo et al. performed three courses of hepatic arterial infusion chemotherapy (HAIC) before stage II ALPPS. The chemotherapeutic agents used included oxaliplatin, fluorouracil, and leucovorin (15). In this case, to facilitate FLR growth and prevent possible tumor progression during the waiting period, TACE was considered due to the rich experience of our center with TACE. Notably, TACE itself can induce hepatic failure, especially in patients with cirrhosis and low FLR (16). However, after a comprehensive evaluation of the patient's preference status, performance status, and recovery of liver function, our MDT team concluded that the risk associated with the TACE procedure in this patient was low. As expected, the patient did not suffer many severe adverse effects after TACE. The absolute kinetic growth increased slightly from 10.00 to 10.62 ml/day. The FLR grew smoothly and finally met the criteria for stage II surgery (5), and the patient successfully underwent a right hepatectomy. Taking them together, interventions that involve localized chemotherapy and chemoembolization appear to be safe and effective in inducing more rapid FLR growth during the interstage period of Mini-ALPPS. In addition, even unsuccessful TACE before ALPPS does not appear to cause the failure of the ALPPS procedure (17). Nevertheless, more evidence is needed to further confirm the safety and risk associated with the use of these additional therapies before or after ALPPS surgery.

Most available reports focus on short- and intermediate-term survival after ALPPS, and data on long-term survival are limited. The reported median survival and median disease-free survival for patients with HCC and MVI undergoing ALPPS are 22 months (range, 3–40 months) and 15 months (range, 5–26 months), respectively (18). Compared with one-stage hepatectomy and PVE, ALPPS could achieve a similar survival benefit. Furthermore, ALPPS could confer better survival benefits than TACE (3, 4, 19, 20). Our case survived 1,922 days following Mini-ALPPS. The long-term survival, in this case, is likely attributable to multidisciplinary treatment and rigorous follow-up, but successful tumor resection was the starting point in all of the cited cases.

The case reported here indicates the importance of damage control during a difficult conventional stage I ALPPS. TACE during the interstage and postoperative periods of this Mini-ALPPS is safe and beneficial. With mini-ALPPS-based multidisciplinary treatment, the patient achieved long-term survival. Nevertheless, some other minimally invasive and effective strategies (such as PVE-based treatments and some modified ALPPS) would be more feasible as the primary solution for such patients today. Not only is the risk of surgery increased by this Mini-ALPPS procedure, but FLR also loses the benefit of rapid hypertrophy.

## Conclusions

Damage control during a difficult conventional stage I ALPPS is important. TACE during the interstage and postoperative periods of this Mini-ALPPS is safe and beneficial. Mini-ALPPS-based multidisciplinary treatment could increase patients’ long-term survival. However, Mini-ALPPS should not be selected as the primary solution for such patients today, as some other minimally invasive and effective strategies are available.

## Data Availability

The original contributions presented in the study are included in the article/Supplementary Material, further inquiries can be directed to the corresponding authors.
